# The effect of interleukin-8 in the early stage after anterior cruciate ligament reconstruction with remnant preservation

**DOI:** 10.1186/s43019-019-0024-0

**Published:** 2020-01-01

**Authors:** Kyung-Ok Kim, Jae Ang Sim, Ji Uk Choi, Beom Koo Lee, Hong Gi Park

**Affiliations:** 1 Gachon Medical Research Institute, Gil Medical Center, Gachon University, 38-13 Dokjeom-ro 3beon-gil, Namdong-gu, Incheon, 21565 Republic of Korea; 2 Department of Orthopaedic Surgery, Gil Medical Center, Gachon University, 21 Namdong-daero 774beon-gil, Namdong-gu, Incheon, 21565 Republic of Korea

**Keywords:** Interleukin-8, Angiogenesis, Joint fluid, Remnant preservation, Anterior cruciate ligament reconstruction

## Abstract

**Purpose:**

We studied the effect of interleukin-8 (IL-8) as the factor for angiogenesis in the joint fluid of remnant-preserved anterior cruciate ligament reconstruction (RP-ACLR).

**Materials and methods:**

We measured 12 cytokines in joint fluid by multiplex assay and assessed the relationship between IL-8 and vascular endothelial growth factor (VEGF) concentrations. The signal intensity and mean sagittal diameter via postoperative magnetic resonance imaging (MRI) scans were evaluated and the stress X-ray image was analyzed at 3, 6, and 12 months after operation.

**Results:**

The IL-8 concentration was highest 3 months postoperatively in those patients who underwent RP-ACLR. Clinical data also showed that the signal intensity and stress radiography of the knee graft were significantly better at the early postoperative stage.

**Discussion:**

Our results show that IL-8 plays an important role in angiogenesis within 3 months after RP-ACLR. This effect yields better recovery after operation. RP-ACLR patients with high knee stability in clinical data were identical to those with high expression of IL-8 in experimental data. Therefore, IL-8 has been shown to help revascularization and ligamentization of the grafted tendon. These results indicate that IL-8 in RP-ACLR is an important factor for angiogenesis after operation. Unfortunately, the relationship of IL-8 and VEGF in vivo has not been studied.

**Conclusion:**

Our results showed that the IL-8 concentration was very high within 3 months after RP-ACLR operation. The increase in concentration of IL-8 over time was consistent with the increase in VEGF concentration. In the IL-8 clinical setting, MRI analysis showed that ACL synovialization and tension were better in patients who underwent the remnant preservation method. In addition, it was shown that RP-ACLR may be advantageous for early anterior stability within 1 year post operation and beneficial for tendon graft in the early stage post operation. Taken together, our findings suggest that IL-8 may contribute to angiogenesis which is helpful for revascularization and ligamentization of the graft tendon in the early stages of RP-ACLR.

## Introduction

Treatment of anterior cruciate ligament (ACL) rupture can lead to instability of the knee joint due to meniscus injury and cartilage damage, and later to post-traumatic arthritis [1, 2]. In the current clinical setting, ACL reconstruction (ACLR) has become a common orthopedic surgical procedure, improving the stability and the function of the damaged knee following an injury [1]. In conventional ACLR, remnant tissues are completely removed in order to prevent collision with the interproximal joint at the end of the extension of the knee joint. However, proper preservation of remnant tissue can help to stabilize the knee joint by preserving the inherent receptive sensory function through the neural reformation of the graft tendon. In addition, the preserving method has many merits such as an improvement of revascularization and a lower incidence of tibial bone tunnel enlargement [2]. Although this procedure is technically difficult and causes notch impingement, efforts to protect the ACL in surgical treatment have been attempted after Schults et al. first described ACL proprioceptive sensory function [3, 4]. Georgoulis et al. [5] reported that preservation of remnant tissue helped to stabilize the knee joint by protecting the inherent receptive sensory function of the grafted ligament. Ochi et al. studied the preservation of nerve components and mechanical receptors in ACL, and the positive effect on a neural regeneration of blood flow supply and grafting by not removing ACL remnant tissue that impedes anterior relaxation [6]. The clinical utility of remnant preservation ACLR has produced good outcomes [5, 7].

We classified 12 factors into five categories as anti-inflammation, pro-inflammation, bone and cartilage destruction, chemokines, and angiogenesis in joint fluid. Interleukin-1 (IL-1) is known to be produced in chondrocytes and synoviocytes and to promote arthritis by producing protease in chondrocytes. Tumor necrosis factor-α (TNFα) is also one of the powerful cytokines that induce cartilage destruction and has been reported to cooperate with IL-1. IL-6 promotes the differentiation of B cells and induces the proliferation and production of antibodies, and is produced by a variety of cells including chondrocytes and synoviocytes [8]. The biological role of IL-6 has been reported to produce pyrogens and acute phase proteins similar to IL-1, and is also known to increase synovial morbidity and destroy cartilage in patients with rheumatoid arthritis [9, 10]. Some studies have reported that synovial fluid IL-10 was higher after ACL injury and ACLR compared to the control knee and was elevated immediately after injury but decreased to more normal levels in chronic lesions [11]. IL-10 is also known to be a modulatory cytokine that plays an active role in antagonizing TNFα in the knee joint environment.

We focused on the role of IL-8 between remnant-preserved and conventional ACLR. IL-8 is a cytokine that is often associated with inflammation and is important in regulating tumor growth, metastasis, and angiogenesis [12–15]. The direct role of IL-8 in angiogenesis has not been studied. According to some studies, IL-8 is known to stimulate vascular endothelial growth factor (VEGF) expression through ERK and PI3K activation in stem cells and endothelial cells [12, 15]. In addition, VEGF induces the expression of IL-8 in vascular endothelial cells and is involved in neutrophil migration [16].

We studied the effect of IL-8 as the factor for angiogenesis in the joint fluid of RP-ACLR. Our hypothesis was that IL-8 may be helpful for postoperative repair due to the role of angiogenesis. Based on biological research and clinical outcome, we analyzed a follow-up period of 18 months postoperatively to investigate whether IL-8 can help in recovery of revascularization and ligamentization after RP-ACLR.

## Materials and methods

### Patient selection

We retrospectively reviewed the charts of 40 consecutive patients who underwent ACLR performed by a single surgeon between March 2010 and February 2014. We performed this study by collecting joint fluid at 3-month intervals in two groups of patients who underwent remnant-preserved ACLR and conventional ACLR. We left half of the ACL tissue when operating the patients. Ninety-five patients consented to this study, and the study design passed a strict examination by the institutional review board. Although 95 patients agreed to this study, most patients did not come to the hospital after 3 months post operation. We evaluated the patients who came to the hospital every 3 months periodically out of 95 patients. Therefore, we selected 20 patients who underwent RP-ACLR and 20 patients who underwent conventional ACLR (Table 1). We obtained joint fluid first in the operating room and then every 3 months in the doctor’s room. The obtained samples were immediately delivered to the laboratory. We centrifuged at 2000 rpm for 10 min to remove debris from the samples. All samples were immediately frozen in 100 μl aliquots and stored at − 80 °C until analyses were performed.

**Table 1:** Demographic data of patients in the two groups

	Remnant-preserved group (*n* = 20)	Conventional group (*n* = 20)
Median age, years (range)	36 (19–56)	33 (19–59)
Gender (male/female)	13/7	17/3
Involved side (right/left)	6/14	13/7
Follow-up period, months (mean ± standard deviation)	13.3 ± 4.6	13.1 ± 4.4

### Multiplex assay

We investigated the concentration of 12 cytokines in the joint fluid between the day of operation and 3 months after operation by multiplex assay. We classified 12 factors into five categories as anti-inflammation (IL-10, IL-11), proinflammation (GM-CSF, IL-6, IL-8), bone (IL-1β, TNFα) and cartilage (IL-17) destruction, chemokines (MCP-1, RANTES, SDF1α + β), and angiogenesis (VEGF). We used HCYTO60K08, HCYTO60K01, HCYP363K01, HCYP262K01, and LXSAHM-1 kits (Millipore, Billerica, MA, USA). We added 200 μl of wash buffer to the plates. After washing, we added 25 μl standards or samples to the appropriate wells, and added 25 μl beads to each well. On the next day, 25 μl of detection antibodies was added to the plates. We added 25 μl streptavidin–phycoerythrin to the plates containing detection antibodies, and added 150 μl sheath fluid into each well. Finally, we read the cytokine/chemokine concentrations in the plates on a Luminex^200^™ (Luminex Corporation, Austin, TX, USA).

### ELISA

IL-8 enhanced endothelial cell survival, proliferation, and neovascularization and regulated angiogenesis; however, the role of IL-8 in ACLR is less well defined. In general, VEGF is known as a signal protein which contributes to vasculogenesis and angiogenesis regardless of the surgical methods. We investigated the VEGF concentration to identify whether IL-8 is related to angiogenesis.

We used K0331216 and K0331132 kits for ELISA (Koma Biotech Inc., Seoul, Korea). We added 200 μl of washing solution to each well. After removing the wash buffer, we added 100 μl of standards or samples in triplicate and incubated at room temperature for at least 2 h. For binding with antibodies, we added 100 μl of 0.5 μg/ml IL-8 and 0.25 μg/ml VEGF and incubated at room temperature for 1 h. The next step was to add 100 μl of the diluted color development enzyme and then we added 100 μl of color development solution and incubated at room temperature for proper color development. To stop the color reaction, we added 100 μl of the stop solution. We used a microtiter plate reader (E max; Molecular Devices) to read the plate at a wavelength of 450 nm.

### MRI analysis

We performed MRI of 27 patients (remnant preservation: 14 cases, conventional: 13 cases), and compared the ACL signal intensity and sagittal diameter after a mean period of 12 months post operation. We used the MRI scan for analysis of the signal intensity and mean sagittal diameter.

### Signal intensity score and sagittal diameter analysis

The ACL signal intensity and mean sagittal diameter were measured on the MRI scan after a mean period of 12 months post operation. The intra-articular portion of the graft was divided into three areas as proximal, mid, and distal. We analyzed the MRI scan of ACL grafts using the method described by Hong et al. [17]. We measured the signal intensity graded from 0 to 3 points (0 = low signal, 1 = increased signal in less than 50%, 2 = increased signal in more than 50%, 3 = diffuse-increased signal). The sum of the three areas points was the score of the graft signal intensity. To quantify the normalized signal intensity of the ACL graft, the signal/noise quotient (SNQ) was calculated using the region of interest (ROI) technique (diameter of the circle was 3.3 mm) with the following equation:

$$ \mathrm{SNQ}=\left(\mathrm{signal}\ \mathrm{of}\ \mathrm{ACL}\ \mathrm{graft}-\mathrm{signal}\ \mathrm{of}\ \mathrm{quadriceps}\ \mathrm{tendon}\right)/\mathrm{signal}\ \mathrm{of}\ \mathrm{background}. $$

The graft sagittal diameter was evaluated in three areas, the same as the signal intensity.

### X-ray stress radiography analysis

Stress radiography was performed using a Telos device. We analyzed stress X-ray images from 17 patients (remnant preservation: 8 cases, conventional: 9 cases) at 3 months post operation. Twenty patients who underwent RP-ACLR and 19 patients who underwent conventional ACLR were analyzed 6 months postoperatively. In addition, 9 patients who underwent RP-ACLR and 17 patients who underwent conventional ACLR were analyzed 12 months postoperatively.

### Statistical analysis

Differences in outcomes were assessed by Student’s *t* test. Data were indicated as the mean ± standard deviation and analyzed using SPSS (SPSS, Inc., an IBM Company, Chicago, IL, USA). Null hypotheses of no difference were rejected if *p* < 0.05. The clinical data were analyzed using the Mann–Whitney test.

## Results

### Cytokine concentration on ACLR

**Fig. 1:**
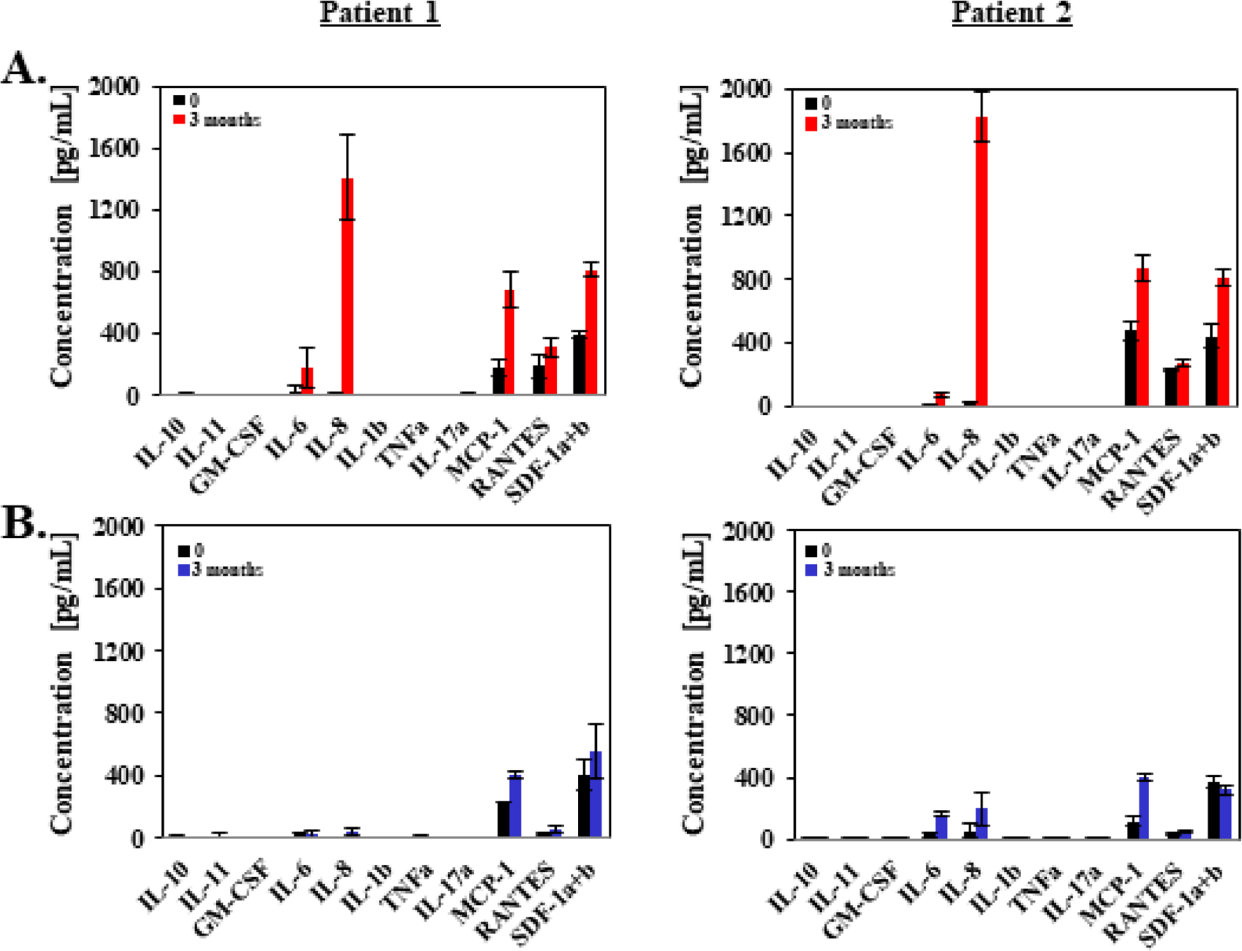
Cytokine concentrations in the joint fluid of the two anterior cruciate ligament reconstruction (ACLR) groups. **a** Remnant-preserved ACLR in two patients. **b** Conventional ACLR in two patients. The time points were the day of operation and 3 months post operation. Data are representative of experiments that were repeated three times. a alpha, b beta, GM-CSF granulocyte–macrophage colony-stimulating factor, IL interleukin, MCP monoctye chemoattractant protein, RANTES regulated on activation, normal T cell expressed and secreted, SDF stromal cell-derived factor, TNF tumor necrosis factor

The purpose of this study was to understand the effect of IL-8 in joint fluid after ACLR. Prior to establishing the role of IL-8, the concentrations of commonly noted cytokines in the joint fluid were measured. We investigated the concentration of 12 cytokines in the joint fluid between the day of operation and 3 months after operation by multiplex assay. We found that the IL-8 concentration was higher 3 months postoperatively in the patients treated with remnant-preserved ACLR Fig. 1a shows 2 of 20 RP-ACLR patients and Fig. 1b shows 2 of 20 conventional ACLR patients. We explored the concentration of IL-8 with and without RP-ACLR every 3 months up to 18 months post operation by multiplex assay and ELISA. In the next experiment, we examined the concentration of cytokines other than IL-8 between the two groups, but there was no difference in the remaining 11 cytokines.

### Comparison of IL-8 concentrations in the two groups

**Fig. 2:**
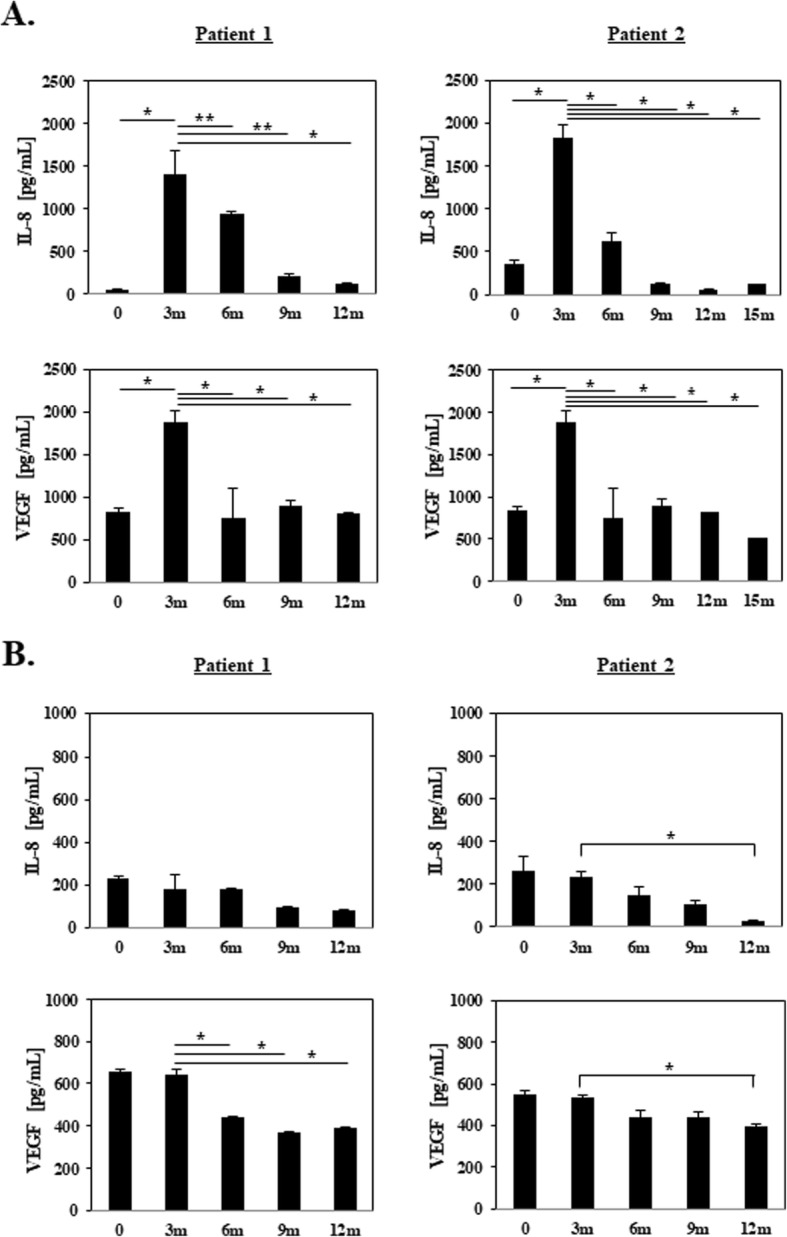
**a** Relationship between IL-8 and VEGF in remnant-preserved ACLR. We selected two patients who underwent remnant-preserved ACLR: IL-8 concentration (top panels) and VEGF concentration (bottom panels). Data expressed as median value; *p < 0.05, **p > 0.05 compared to 3 months postoperatively by Student’s *t* test. Data are representative of experiments that were repeated three times. **b** Relationship between IL-8 and VEGF in conventional ACLR. We selected two patients who underwent conventional ACLR. IL-8 concentration on a trimonthly basis (top panels) and VEGF concentration on a trimonthly basis (bottom panels). Data were expressed as median value; *p < 0.05 compared to 3 months postoperatively by Student’s *t* test. Data are representative of experiments that were repeated three times. ACLR anterior cruciate ligament reconstruction, IL-8 interleukin-8, m months, VEGF vascular endothelial growth factor

**Fig. 3:**
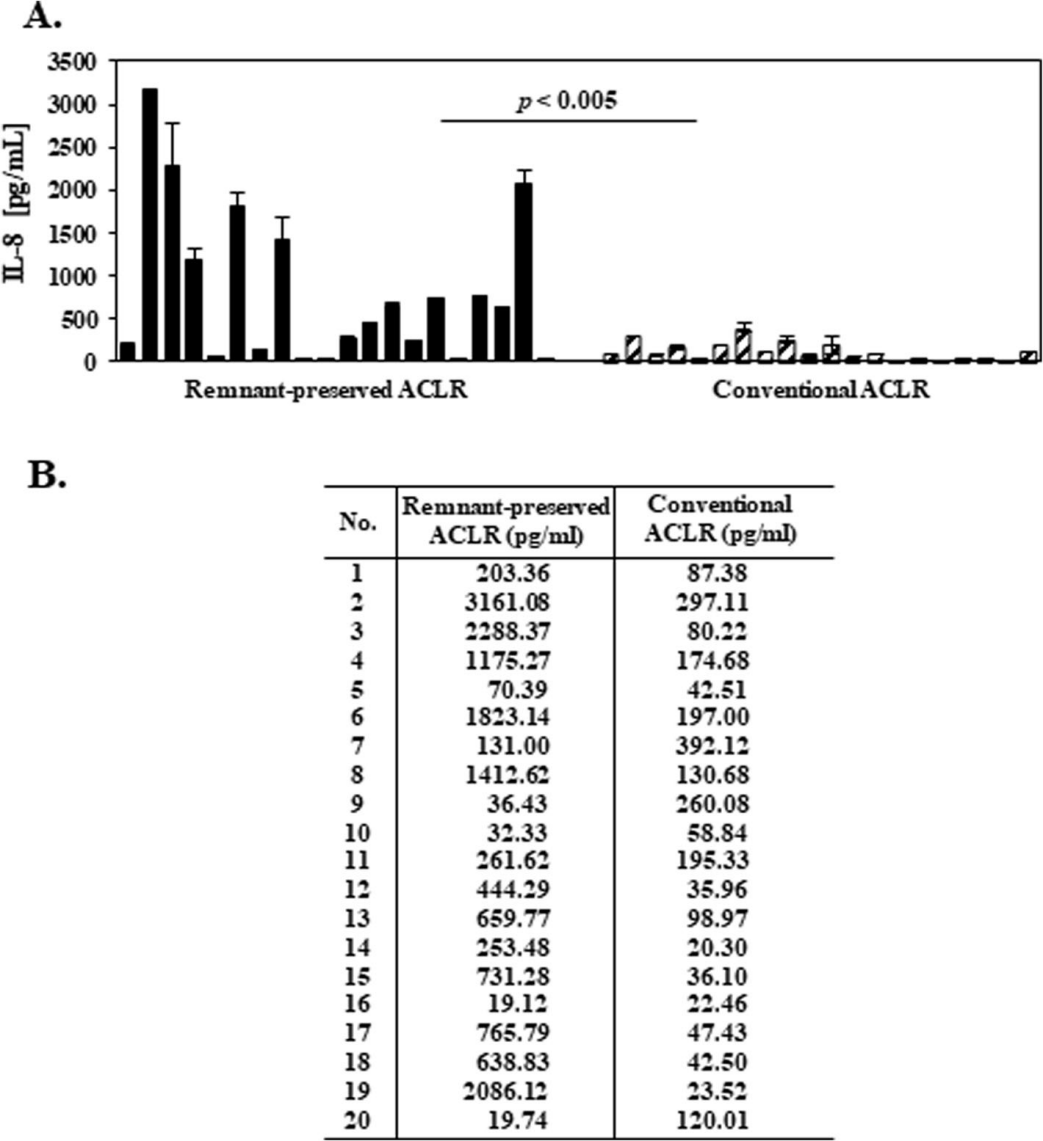
**a** IL-8 concentration between remnant-preserved ACLR and conventional ACLR in 20 patients, respectively. **b** Average of two groups that repeated independent experiments three times. Differences were statistically significant, *p* < 0.005 comparing two groups by Student’s *t* test. ACLR anterior cruciate ligament reconstruction, IL-8 interleukin-8

In 14 of the 20 patients who underwent RP-ACLR, the concentration of IL-8 robustly increased 1.7–230 times within 3 months compared to the day of operation (Fig. 2a). The IL-8 concentration decreased to a basal level mostly after 6 months (data not shown). The IL-8 concentration increased in only 5 of 20 patients who underwent conventional ACLR, over the first 3 months, and the increased concentration was very low at 1.1–2.4 times (Fig. 2b). The IL-8 concentration did not increase in most subjects treated with conventional ACLR during the 3 months that is the initial activation point for blood vessel formation. Figure 3 shows the IL-8 concentration between remnant-preserved ACLR and conventional ACLR of 20 patients, respectively. The mean IL-8 concentration in 20 patients who underwent RP-ACLR was 801.70 pg/ml and in 20 patients who underwent conventional ACLR was 117.41 pg/ml. The concentration of IL-8 was statistically significantly different in patients between 3 months and the day of operation in the two groups.

### Relationship between IL-8 and VEGF in the two groups

The relationship between IL-8 and VEGF in the two groups is shown in Fig. 2a, b. Our results indicated that IL-8 increased at the early stage of 3 months after remnant-preserved ACLR. The VEGF concentration increased over time with the remnant preservation method 3 months postoperatively, and then gradually decreased until 9 and 15 months (Fig. 2a). The VEGF results were consistent with the IL-8 results on a trimonthly basis. With the conventional method, the VEGF concentration did not increase 3 months postoperatively but decreased over the observation period trimonthly. Taken together, our results indirectly suggest that IL-8 may be helpful for vasculogenesis and angiogenesis with VEGF at the early postoperative stage in the remnant preservation method.

### MRI analysis of the two groups

**Fig. 4:**
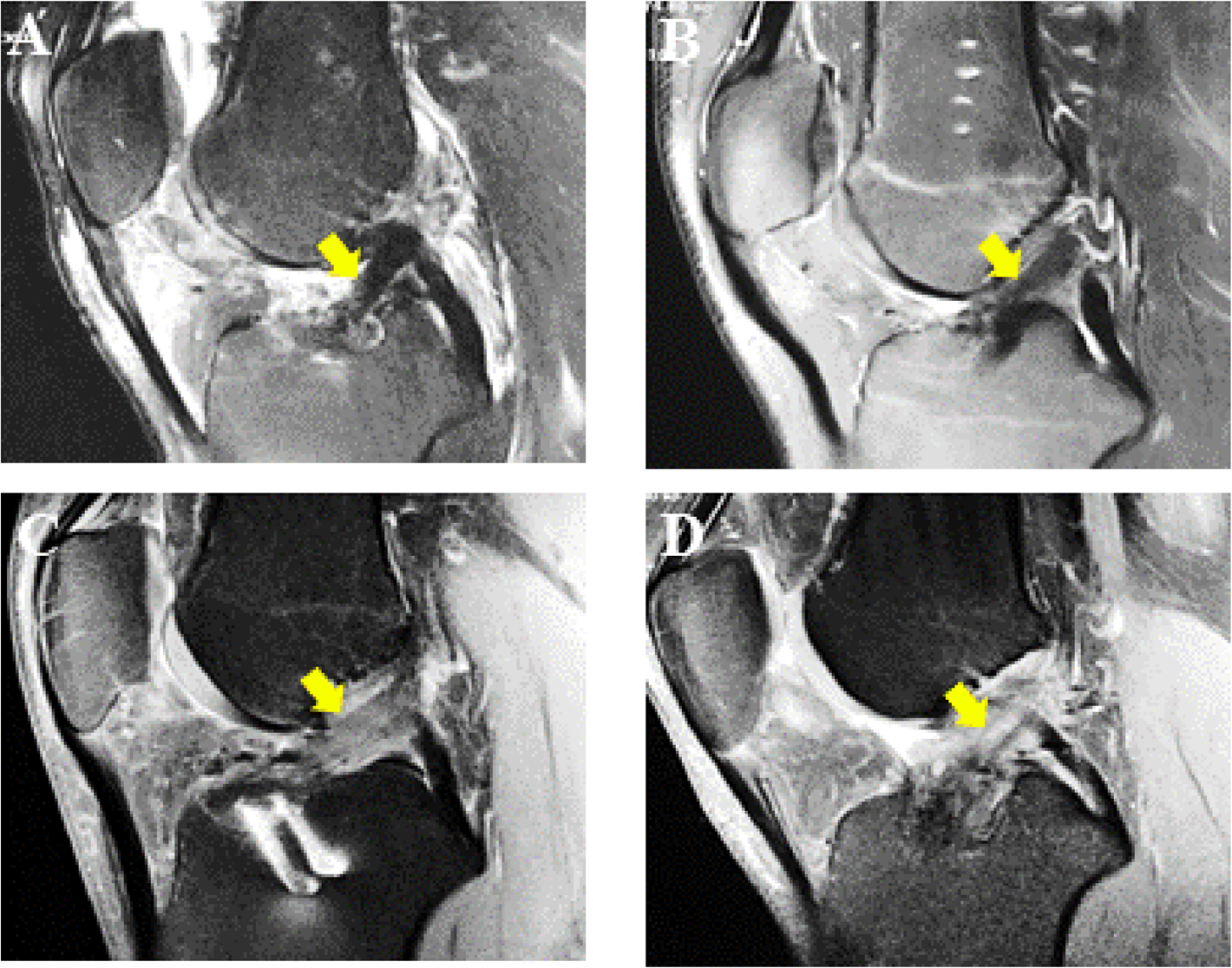
MRI scans from the two groups: **a** 30-year-old male and **b** 19-year-old male who underwent remnant-preserved ACLR; and **c** 21-year-old male and **d** 22-year-old male who underwent conventional ACLR. MRI scans were taken at a mean period of 12 months. Yellow arrows indicate reconstructed ACL grafts. ACLR anterior cruciate ligament reconstruction, MRI magnetic resonance imaging

Clinical data (Tables 1, 2 and 3 and Fig. 4) were analyzed among the same patients (RP-ACLR: 20 patients, conventional ACLR: 20 patients) in parallel with the experimental data (Figs. 1, 2 and 3). The MRI scan was taken a mean period of 12 months after operation. We used the MRI scan for analysis of the signal intensity and mean sagittal diameter. The MRI analysis showed that ACL synovialization and tension were better in patients who underwent the remnant preservation method.

**Table 2:** Comparison of signal intensity and sagittal diameter in the two groups

	Signal intensity	Sagittal diameter (mm)
Remnant-preserved group (*n* = 14)	2.50 ± 0.69	9.88 ± 0.41
Conventional group (*n* = 13)	5.07 ± 0.92	10.11 ± 0.54
*p* value	0.048	0.616

**Table 3:** Comparison stress X-ray data 3, 6, and 12 months postoperatively in the two groups

Postoperatively	Remnant-preserved group	Conventional group	*p* value
3 months	(*n* = 8)	(*n* = 9)	
30° flexion (mm)	0.46 ± 0.19	3.39 ± 0.28	< 0.001
90° flexion (mm)	0.79 ± 0.25	3.19 ± 0.21	< 0.001
6 months	(*n* = 20)	(*n* = 19)	
30° flexion (mm)	1.15 ± 0.25	2.96 ± 0.28	< 0.001
90° flexion (mm)	0.76 ± 0.19	2.73 ± 0.25	< 0.001
12 months	(*n* = 9)	(*n* = 17)	
30° flexion (mm)	1.47 ± 0.40	2.87 ± 0.34	0.018
90° flexion (mm)	0.89 ± 0.25	2.74 ± 0.30	< 0.001

Data presented as mean ± standard deviation. Stress radiography performed using a Telos device

The signal intensity analysis was significantly higher in the conventional ACLR patients than in the RP-ACLR patients, but there was no significant difference in sagittal diameter analysis between the two groups (Table 2). Our study demonstrates that RP-ACLR may be more advantageous than conventional ACLR for early anterior stability within 1 year after operation in the clinical setting.

### Stress X-ray analysis

We analyzed stress X-ray images from 17 patients (remnant preservation: 8 cases, conventional: 9 cases) at 3 months post operation. Twenty patients who underwent RP-ACLR and 19 patients who underwent conventional ACLR were examined 6 months postoperatively. In addition, 9 patients who underwent RP-ACLR and 17 patients who underwent conventional ACLR were analyzed 12 months postoperatively. RP-ACLR was significantly more stable than conventional ACLR 3, 6, and 12 months postoperatively (Table 3). These analyses suggest that RP-ACLR may be beneficial for tendon graft in the early stage post operation.

## Discussion

The most important finding of the current study was that the IL-8 concentration was higher for RP-ACLR than for conventional ACLR, especially at the early stage after the operation. We also found correlation of IL-8 and the VEGF concentration over time.

ACL rupture is one of the most common injuries in orthopedic sports trauma. Conventional ACLR removes ligament debris and reconstructs grafts to prevent the development of Cyclops’ lesions. ACLR of remnant preservation is technically more difficult and cumbersome than the conventional surgical method. Nevertheless, method of RP-ACLR, remaining self-organization is help for tissue repair. Since remnant tissue has a mechanical receptor, it has an inherent sensory function; and because blood flow is maintained, preservation of the remnant tissue may help restore knee position sensation and ligamentization of the graft after ACLR, which may affect functional outcome [18–20]. In addition, it has many advantages of biomechanical properties such as an improvement of revascularization, preservation of the proprioceptive neural elements, and a lower incidence of tibial bone tunnel enlargement [2, 18, 21, 22]. It is useful for restoration of function after operation, ligamentization of the grafted tendon, and improvement of stability. The remnant tissue is known to have a biomechanical function to resist anterior displacement of the tibia in addition to the biological function. Therefore, it is believed that the preservation of remnant tissue improves the mechanical stability immediately after the operation. Clinically, remnant preservation of ACLR has produced good outcomes, but evidence of biological function is insufficient. Zhang et al. [21] reported that remnant preservation in ACLR may accelerate cell repopulation and revascularization in the graft, resulting in acceleration of graft remodeling and early restoration of the mechanical properties of the graft. Ahn et al. [19] described that although postoperative MRI showed no significance between preservation and conventional methods, ACLR with remnant preservation with femoral tensioning is a good surgical option for clinical outcomes of knee stability and second-look arthroscopy. Relatively, Lee et al. [23] reported that remnant preservation in ACLR can resist tibial tunnel enlargement but this method does not affect the short-term clinical outcomes of ACLR. In a short-term study, Hong et al. [24] reported that ACLR with remnant preservation had no evident advantages in clinical outcomes compared with the conventional method.

In previous basic animal studies, remnant tissue improved ACL graft ligamentization and graft-tunnel enlargement [2]. However, other studies showed that remnant-preserved ACLR did not improve graft ligamentization [19, 25]. Therefore, the effects of remnant preservation are still controversial [21, 23, 24].

We classified 12 factors into five categories — anti-inflammation, pro-inflammation, bone and cartilage destruction, chemokines, and angiogenesis in joint fluid — and measured the concentrations. Among these, IL-8 was robustly increased at an early stage after the operation. The concentration was increased only in the method of remnant-preserved ACLR. IL-8 is a pre-inflammatory C–X–C chemokine that was previously characterized as a major agent of neutrophil recruitment and activation. In the synovial membrane, IL-8 is constitutively secreted by synovial macrophages, while fibroblast-like synoviocytes produce IL-8 only in the presence of agents such as IL-1α, IL-1β, TNFα, and LPS [26]. IL-8 has also been found to play a pivotal role in angiogenesis, one of the main mechanisms for the maintenance and persistence of chronic synovial inflammation [27, 28].

The most import finding of this study is that IL-8 may contribute to angiogenesis at the early stage after RP-ACLR. We observed that IL-8 was promising in helping revascularization and ligamentization of the grafted tendon. We believe that studies should be conducted to more accurately demonstrate the relationship between IL-8 and angiogenesis.

Our results have limitations. One of these limitations is that the follow-up time is less than 2 years. The other limitation is that the results has not been fully experimentally investigated. Therefore, the future plan is to more clearly explain the role of IL-8 through animal experiments.

## Conclusion

Our results showed that the IL-8 concentration was very high within 3 months after RP-ACLR operation. The increase in IL-8 concentration over time was consistent with the increase in VEGF concentration. In the IL-8 clinical setting, MRI analysis showed that ACL synovialization and tension were better in patients who underwent the remnant preservation method. In addition, it was shown that RP-ACLR may be advantageous for early anterior stability within 1 year post operation and beneficial for tendon graft in the early stage post operation. Taken together, our findings suggest that IL-8 may contribute to angiogenesis which can be helpful for revascularization and ligamentization of the graft tendon in the early stages of RP-ACLR.

## Data Availability

Data sharing is not created or analyzed during the current study and does not apply to articles. Please contact the author for data requests.

## Abbreviations

ACL: Anterior cruciate ligament
ACLR: Anterior cruciate ligament reconstruction
IL-8: Interleukin-8
RP-ACLR: Remnant-preserved anterior cruciate ligament reconstruction
VEGF: Vascular endothelial growth factor
